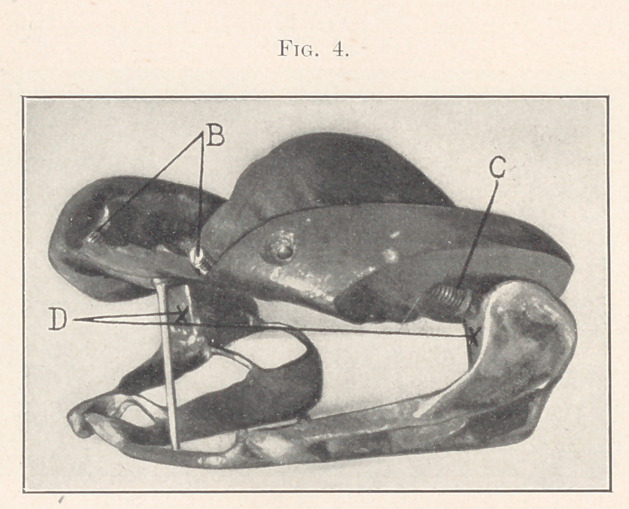# Jumping the Bite

**Published:** 1903-08

**Authors:** George B. Terrell

**Affiliations:** New York


### JUMPING THE BITE.[1]

BY GEORGE B. TERRELL, D.D.S., NEW YORK.

THERE has been much discussion relative to jumping the bite, as to whether the shape of the lower jaw is changed, or the condyles move forward or backward and make for themselves a new point from which to swing.

I present to you the problem again in the case of a girl fourteen years of age, where the lower jaw protruded so that the teeth bit entirely outside of the upper teeth, as you can see by an inspection of the casts (Fig. 1); and where by the use of appliances the arch of the upper jaw was expanded laterally, and then, apparently, the whole lower jaw was forced backward until the teeth bit within the upper arch (Fig. 2).

---

[1] Read before The New York Institute of Stomatology, April 7, 1903.

Fig. 1.

Fig. 2.

FIG. 3.

FIG. 4.

Unfortunately, no photograph of the patient was taken at the beginning, but this photograph, which was taken a few days ago, will show you the completed case, and by comparing the models you can picture to yourselves the profile as it was.

You will also observe by the cast that a bicuspid on each side of the upper jaw is missing.

The first step was to expand the upper arch and push out individual teeth on the sides. This was accomplished with a rubber plate (Fig. 3) split through the middle to within about one-quarter of an inch of the back edge, where it was strengthened with a piece of wire embedded in the rubber. Towards the front, embedded in the rubber and spanning the split, was a screw (A) for spreading the plate. Where teeth were to be pushed out the plate was made thicker, a hole bored opposite the tooth, tapped, and a small screw (B) of German silver turned in. The patient was given a small screw-driver and wrench and directed to turn the screws out against the teeth, and the screw spanning the split a little every day. On account of the interlocking of the upper incisors, it was left to the next plate to push these out.

The next step was to move the upper jaw forward or the lower jaw backward, or both, so as to get the true bite. To do this a crib of German silver wire was fitted around the bicuspid and second molar on each side of the upper jaw, and attached to the crib on the outside opposite the first molar, pointing backward and a little downward, were two tubes, one on each side, about three-eighths of an inch long, which were tapped and screws (C) with good-sized heads fitted to them. Over the crib a plate (Fig. 4) was vulcanized to fit the roof of the mouth, with sufficient thickness of rubber over the ends of the molars and bicuspids to open the bite and allow the upper front teeth to pass over the ends of the lower teeth, and with a little hood of vulcanite over the heads of the screws to prevent them from irritating the insides of the cheeks.

For the lower jaw a crib was made of German silver wire and fitted to the first bicuspid and first molar of each side, and soldered to a heavy wire running around the inside of the arch; and a narrow strip of plate was soldered on top of the molar crib to prevent its jamming down between the teeth. Then, on the outside of the crib an inclined plane (D) was soldered on each side as close to the molars as possible, and so facing that when the jaws were closed the heads of the screws in the upper plate came in contact with the surface of the inclined plane. Various positions were tried, but these were found to be the best.

The patient was directed to turn the screws out about a half-turn every day, and report in a couple of weeks. This was done until it was found that the teeth or the jaws had started to move.

As the patient lived at a considerable distance, it was impossible for her to come to the office as often as she should have done, and it made corrections in the construction of the appliances necessarily slow.

It was discovered after awhile that the constant rubbing of the screw-heads against the plane turned them in again. This was obviated by melting into the tubes a little resin, which, when cold, held the screws fast. Every time the screws were turned a heated iron was held against the heads to soften the resin.

Then, again, it was found that the edge of the vulcanite hood over the screw-heads was too hard, and pinched the cheeks against the apparatus in the lower jaw, so velum rubber was substituted for the hard rubber.

The appliance was worn continually except when eating, and could be taken out and cleansed, and no injury has been done to the teeth. The teeth and appliance have been under perfect control at all times.

The patient is at present wearing small retaining plates to hold the teeth out, the overbite of the upper front teeth keeping the jaws in place. There is not yet a perfect occlusion, but, rather than grind off points on the molars that come in contact, it is thought best to let the teeth grind to their own occlusion.

## Figures and Tables

**Fig. 1. f1:**
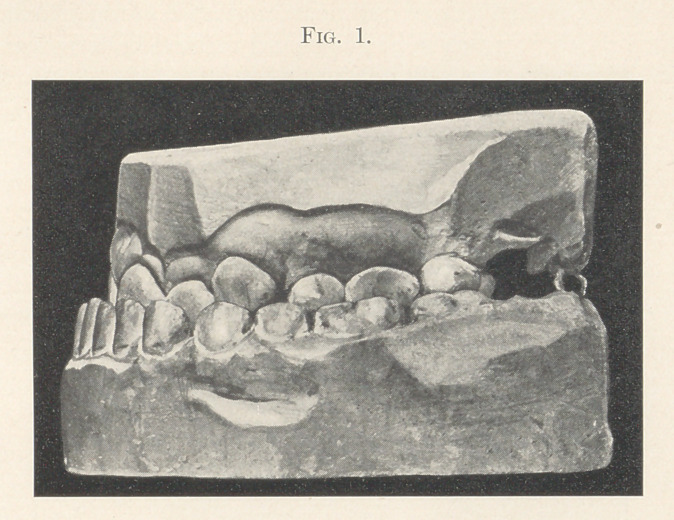


**Fig. 2. f2:**
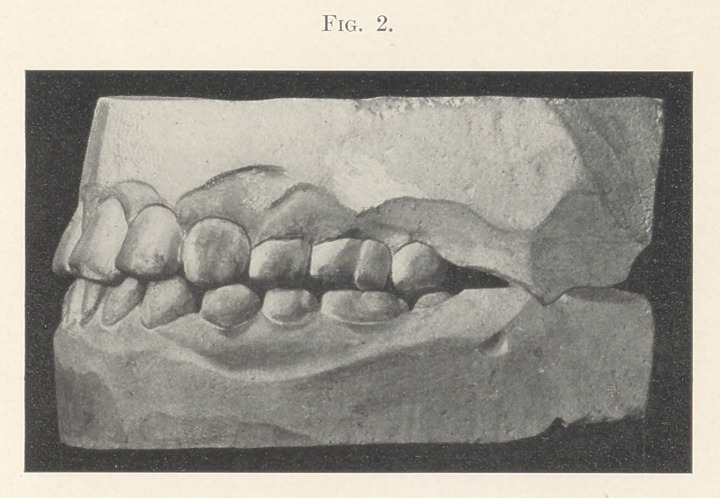


**Fig. 3. f3:**
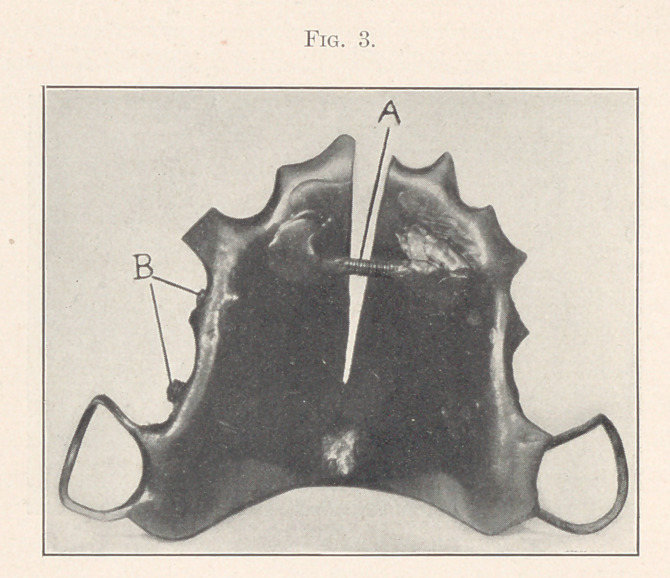


**Fig. 4. f4:**